# A novel imaging method for correlating 2D light microscopic data and 3D volume data based on block-face imaging

**DOI:** 10.1038/s41598-017-03900-9

**Published:** 2017-06-16

**Authors:** Yuki Tajika, Tohru Murakami, Keiya Iijima, Hiroki Gotoh, Maiko Takahashi-Ikezawa, Hitoshi Ueno, Yuhei Yoshimoto, Hiroshi Yorifuji

**Affiliations:** 1 Department of Anatomy, Graduate School of Medicine, Gunma University. 39-22 Showa-machi 3-chome, Maebashi, Gunma, 371-8511 Japan; 2 Department of Neurosurgery, Graduate School of Medicine, Gunma University. 39-22 Showa-machi 3-chome, Maebashi, Gunma, 371-8511 Japan; 3 Laboratory of Sericulture and Entomoresources, Graduate School of Bioagricultural Sciences, Nagoya University. Furo-cho, Chikusa-ku, Nagoya, 464-8601 Japan; 4 Department of Occupational Therapy, Graduate School of Health Science, Gunma University. 39-22 Showa-machi 3-chome, Maebashi, Gunma, 371-8511 Japan

## Abstract

We have developed an imaging method designated as correlative light microscopy and block-face imaging (CoMBI), which contributes to improve the reliability of morphological analyses. This method can collect both the frozen sections and serial block-face images in a single specimen. The frozen section can be used for conventional light microscopic analysis to obtain 2-dimensional (2D) anatomical and molecular information, while serial block-face images can be used as 3-dimensional (3D) volume data for anatomical analysis. Thus, the sections maintain positional information in the specimen, and allows the correlation of 2D microscopic data and 3D volume data in a single specimen. The subjects can vary in size and type, and can cover most specimens encountered in biology. In addition, the required system for our method is characterized by cost-effectiveness. Here, we demonstrated the utility of CoMBI using specimens ranging in size from several millimeters to several centimeters, i.e., mouse embryos, human brainstem samples, and stag beetle larvae, and present successful correlation between the 2D light microscopic images and 3D volume data in a single specimen.

## Introduction

Light microscopy has been used in biology to perform morphological analysis since era of van Leeuwenhoek in the 17th century^1^. Biological samples are generally processed into thin sections, and labeled with chromophores, fluorophores, and antibodies for yielding various types of 2-dimensional (2D) information regarding both anatomy and the distribution of molecules. However, conventional light microscopy is unsuitable for obtaining 3-dimensional (3D) anatomy or determining the 3D distribution of molecules as it generally uses thin sections which have limited information about depth, and lose information about the original position within the specimen^2^. To obtain sections maintaining positional information about the origin in the specimen, we developed a system and method for combining cryostat sectioning and block-face imaging. Our method allows us to obtain both 2D light microscopic data using sections and 3D volume data of serial block-face images from a single specimen, and is named correlative light microscopy and block-face imaging (CoMBI). The CoMBI method involves 3D imaging by serial block-face imaging, which is a technique for reconstructing 3D images from a series of block-face images. Some laboratories have developed apparatus for obtaining serial block-face images from frozen blocks or paraffin-embedded tissue, and reconstructed 3D images from various specimens, such as mouse and zebrafish embryos^3^, rat knee joint^4^, whole adult mouse^5, 6^, and equine ovary^7^. However, these types of apparatus are not widely used. This may be because the sections cannot be utilized for further microscopic analyses as they are discarded as debris during block-face imaging. In addition, the high cost and low availability of the apparatus may hinder the widespread adoption of serial block-face imaging. We have developed the apparatus for CoMBI using readily obtainable components at low cost, and have provided all information, including the component list, code, and drawings, so that our CoMBI method is endowed with the widespread adoption.

Here, we show the applications of our CoMBI system observing a wide variety of biological specimens ranging in scale from several millimeters to several centimeters, such as mouse embryos, human brainstem samples, and stag beetle larvae. The use of frozen blocks of these specimens allowed us to obtain both frozen sections and serial block-face images. Frozen sections retain information about the original position in the specimen, and can be used for 2D microscopy followed by histochemistry or immunofluorescence. Serial block-face images can be used as 3D volume dataset for anatomical analysis by reconstruction of arbitrary planes and volume rendering images. As a result, successful correlations between 2D light microscopic data and 3D volume data of serial block-face images within single specimens are shown.

## Results

### The CoMBI system

**Figure 1:**
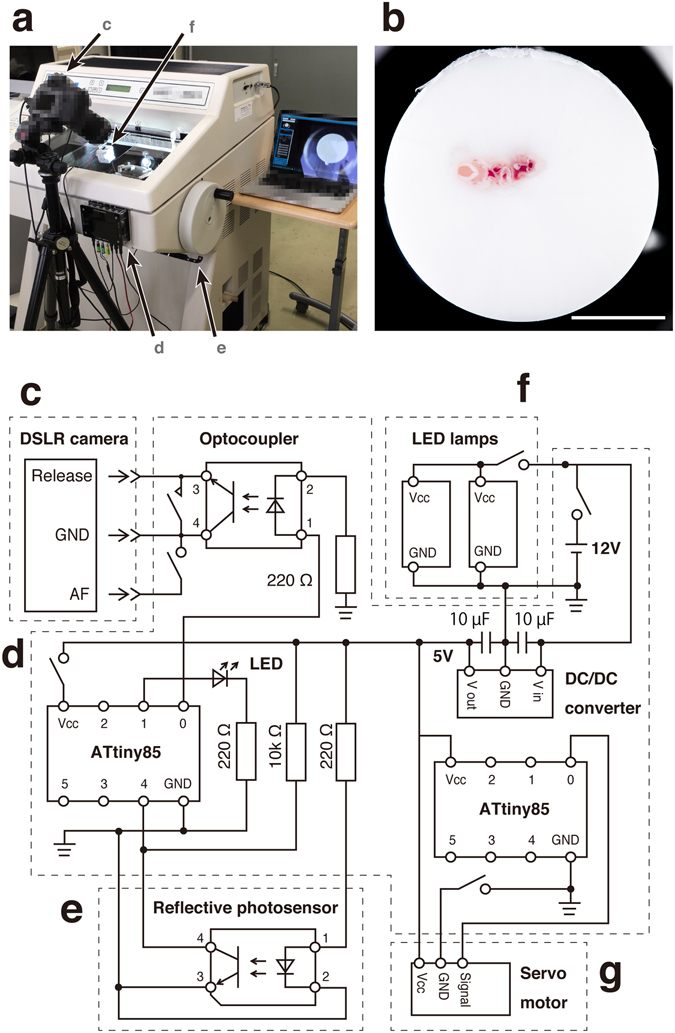
The CoMBI system. (**a**) Photograph of the system. The camera (**c**) is placed in front of the cryostat. The controller (**d**) is attached to the front panel of the cryostat. The sensor (**e**) is attached close to the cryostat handle to detect the handle position. The illumination (**f**) is set in the chamber. (**b**) An example of the block-face image shows the clear outer circumference. (**c**–**g**) Circuit of the system: camera (**c**), controller (**d**), sensor (**e**), illumination (**f**), cleaning brush (**g**). An ATtiny85 microcontroller (left) receives signals about the position of the cryostat handle from the sensor (**e**), and triggers release of the camera shutter (**c**) using an optocoupler. The illumination (**f**) consists of two LED lamps on the door to illuminate the block-face orthogonally. The cleaning brush (**g**) consists of a servomotor and three brushes attached to the back of the knife holder to clean the block-face during imaging. Bar in b: 5 mm.

The key components of the CoMBI system are a cryostat and digital single lens reflex (DSLR) camera (Fig. 1a and c). The camera obtains images of every block-faces every time the cryostat slices the block. The obtained serial block-face images are used as 3D volume data, and reconstructed into volume rendering images (Fig. 1b, Supplementary Videos S1 and S2). The cryostat can also be used to obtain standard frozen sections, which can be used for further 2D microscopic analysis (Supplementary Video S3). The additional components include the controller (Fig. 1d, Supplementary Figs S1 and S2), sensor (Fig. 1e, Supplementary Figs S1 and S3), illumination (Fig. 1f, Supplementary Figs S1 and S4), and cleaning brush (Fig. 1g, Supplementary Fig. S4 and Video S4). The controller determines the specimen position by signals from the sensor adjacent to the cryostat handle, and sends the shutter-release signals to the camera, so that the camera automatically obtain the block-face images at the same specimen position after every sectioning. The time required for acquisition of serial images ranged from 73 to 158 minutes per specimen in this study (Table 1). Each image takes approximately 6 seconds and therefore the duration for imaging varies depending on the size of the specimen and the thickness of sections. The frozen blocks for CoMBI are cylindrical in shape, 14 or 26 mm in diameter, and 25 mm in maximum height (Supplementary Fig. S5). This block size is sufficient to include most specimens for light microscopy encountered in the biological laboratory, as microscopy specimens are generally smaller than a glass slide with a short side length of 26 mm or a cryostat specimen holder with a diameter of 30 mm.

**Table 1:** Acquisition time and data volume.

Specimen	No. of images	Thickness (µm)	Time (min)	Format	Volume (GB)
Mouse E10.5 (Fig. 2)	565	10	84	RAW	7.86
Mouse E10.5 (Fig. 3)	503	10	73	RAW	7.07
Human brainstem (Fig. 4)	1275	20	158	JPEG	10.14
Stag beetle larva (Fig. 5)	1064	20	154	RAW	34.05

As the system takes photographs of moving objects, there is some concern regarding motion blur and shifts between images. The two LED lamps prevent motion blur by giving sufficient illumination, and shortening the required exposure time. The cleaning brush contribute to eliminating shifts between images. The shifts can be eliminated by image registration using ImageJ (Supplementary Video S5). Successful registration was achieved by using frozen blocks with a cylindrical shape and the cleaning brush. Frozen blocks were produced by a procedure that provides an identical outer circumference in every image (Supplementary Fig. S5). The cleaning brush sweeps the debris away from the block to maintain a clear outer circumference of the block (Supplementary Video S4). The image registration program recognizes the shift between images based on the identical and clear outer circumference. The resultant serial block-face images can be reconstructed into 3D images, and the frozen sections can be stained and observed by microscopy, as described in the Methods.

### Mouse embryos

**Figure 2:**
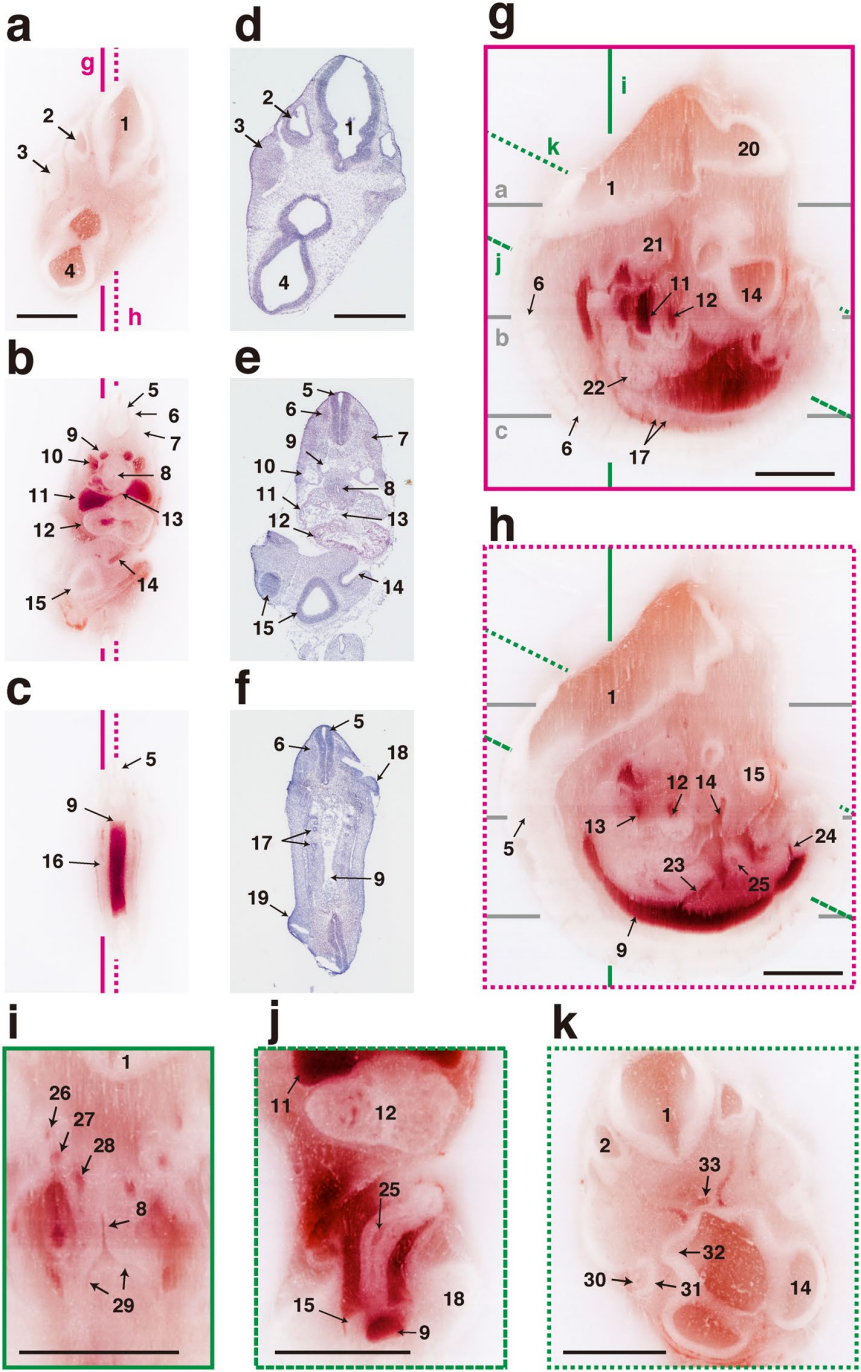
Correlation between 2D images and MPR images of E10.5 mouse embryo. A mouse embryo was imaged by the CoMBI system and shown as MPR images. (**a**–**c**) Block-face images. (**a**–**c**) Frozen sections were collected at the positions of (**a**–**c**). (**g**,**h**) The sagittal planes were made by MPR from 565 block-face images. The nervous, cardiovascular, and urogenital systems are shown. (**i**) Reconstructed coronal planes of the thorax show the respiratory system and vascular system. (**j**) Reconstructed plane of the abdomen shows gastrointestinal tract. (**k**) Reconstructed plane of the head shows sensory organs. 1: myelencephalon, 2: otic vesicle, 3: trigeminal ganglion, 4: diencephalon, 5: neural tube, 6: spinal ganglion, 7: somite, 8: trachea, 9: dorsal aorta, 10: anterior cardinal vein, 11: atrium, 12: ventricle, 13: foramen ovale, 14: olfactory pit, 15: telencephalon, 16: urogenital ridge, 17: mesonephron, 18: forelimb bud, 19: hindlimb bud, 20: mesencephalon, 21: mandible, 22: liver, 23: superior mesenteric artery, 24: umbilical artery, 25: intestine, 26: 3rd aortic arch, 27: 4th aortic arch, 28: 6th aortic arch, 29: bronchus, 30: lens, 31: retina, 32: optic stalk, 33: Rathke’s pocket. Bars: 1 mm. Note that labels 5, 6, 9, 11–14, and 17 indicate structures on lines b and c for correlation between H & E stained sections (**e**,**f**) and MPR images (**g**,**h**).

**Figure 3:**
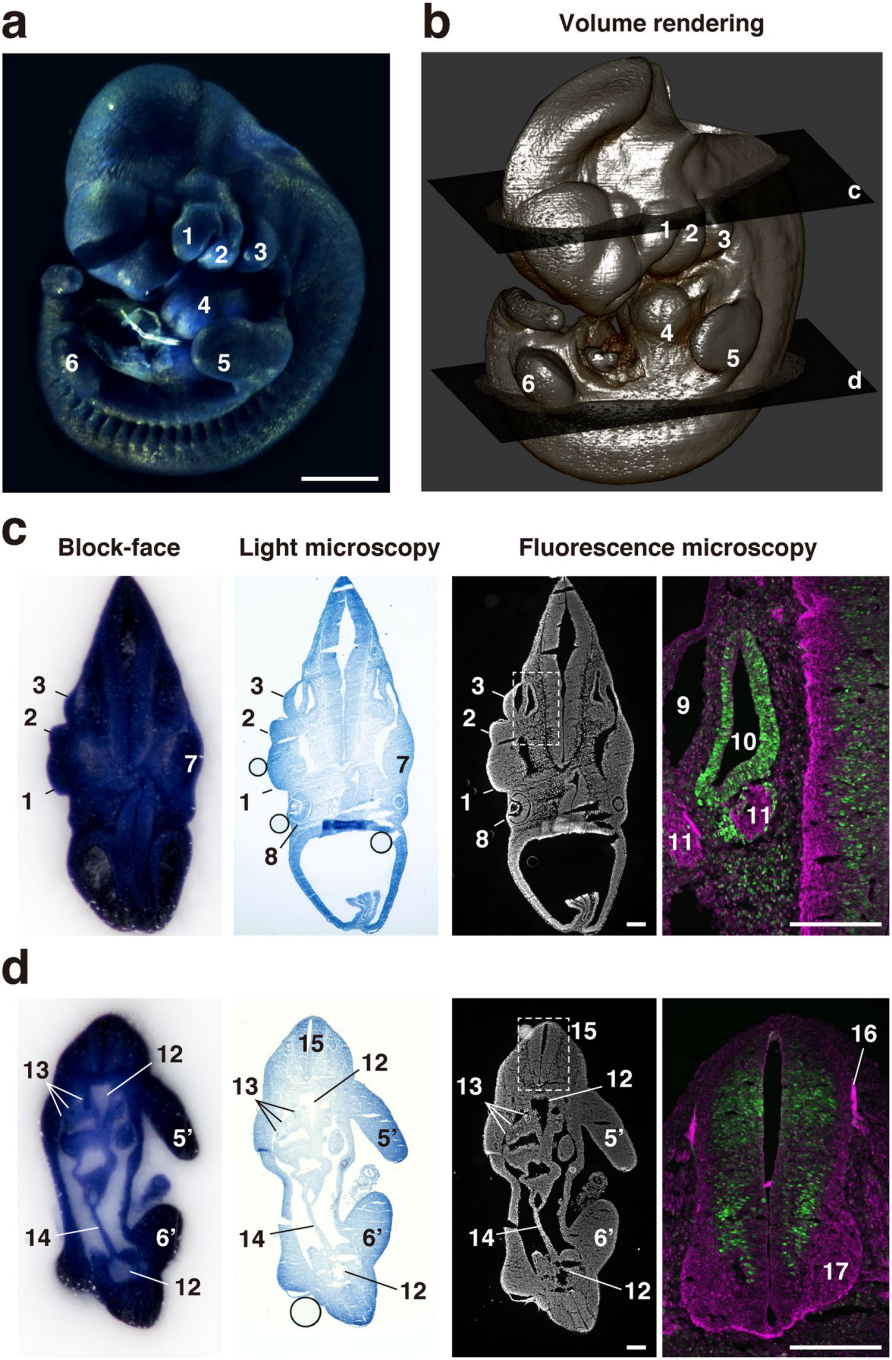
Correlation between 2D images and volume rendering image of E10.5 mouse embryo. (**a**) An E10.5 mouse embryo was prestained with hematoxylin and examined using the CoMBI technique. (**b**) Volume rendering image was reconstructed from 503 block-face images to show the surface structures of the mouse embryo. (**c**,**d**) At the planes c and d in (**b**), frozen sections were collected and fluorescently labeled for nuclei, cyclin D1, and VAMP2. The block-face images are shown in the rightmost column. The sections were examined by light microscopy, and showed nuclei prestained with hematoxylin. The sections were also examined by fluorescence microscopy, and showed nuclei labeled with DAPI. Distributions of VAMP2 (**a** marker of synaptic vesicles, green) and cyclin D1 (cell cycle marker, magenta) are shown at higher magnification. 1: maxillary process, 2: mandibular arch, 3: hyoid arch, 4: heart, 5: left forelimb, 5′: right forelimb, 6: left hindlimb, 6′: right hindlimb, 7: trigeminal ganglion, 8: eye, 9: primary head vein, 10: otic vesicle, 11: facioacoustic ganglion complex, 12: dorsal aorta, 13: mesonephros, 14: mesentery, 15: neural tube, 16: dorsal root, 17: ventral horn. Bars in a: 1 mm, c, d: 200 µm.

The mouse is a commonly used experimental animal, and its embryos are often subjected to morphological analyses. First, we verified the quality of the 3D volume dataset obtained by the CoMBI system, using a fresh-frozen mouse embryo on embryonic day 10.5 (E10.5) (Fig. 2). The block-face images (Fig. 2a–c) and the reconstructed images created by multiple planar reconstruction (MPR, Fig. 2g–k) allowed observation of various organ systems, including the cardiovascular (Fig. 2b,g,h and i), gastrointestinal (Fig. 2g,h and j), respiratory (Fig. 2b and i), urinary (Fig. 2c,g and j), and nervous systems (Fig. 2a–c,g and k). Our system allowed to collect frozen sections on glass slides, stain with hematoxylin and eosin (H&E), and observe by light microscopy (Fig. 2d–f). These sections retained the information about the original positions within the specimen (lines a–c in Fig. 2g and h). Thus, the microscopic images can be correlate with block-face images and MPR images. Correlation between 2D microscopic data and 3D volume data is useful to understand structures in each image data. For example, the position of foramen ovale could be defined in the 2D microscopic image by observation from multiple directions using the MPR image as well as block-face image (“13” in Fig. 2b,e and h). Conversely, mesonephron could be identified in MPR image with a help of microscopy using H&E-stained section (“17” in Fig. 2f and g). Next, the mouse embryo on E10.5 was observed in both volume rendering image and 2D fluorescent microscopic image (Fig. 3). The embryo was prestained with hematoxylin and imaged by the CoMBI system (Fig. 3a, Supplementary Video S2). 3D reconstruction was performed by volume rendering, and showed the fine surface structure of the embryo (Fig. 3b, Supplementary Video S2). The stain enhanced the color of the embryo body, and provided better contrast between the embryo and the mounting media (Fig. 3c and d, “Block-face”), resulting in fine surface rendering (Fig. 3b). Frozen sections were collected in planes c and d in Fig. 3b, and subjected to immunostaining and microscopy. Transmitted light microscopy showed organ structures more clearly than block-face images (Fig. 3c and d, “Light microscopy”). The frozen sections were immunostained for vesicle-associated membrane protein-2 (VAMP2, a marker of synaptic vesicles), and cyclin D1 (a marker of the cell nuclei in the G1 phase of the cell cycle). Fluorescence microscopy visualized the 2D distribution of these molecules in the frozen sections (Fig. 3c and d, “Fluorescence microscopy”). These 2D data retained the 3D positional information in the embryo body, indicating the successful correlation between 3D and 2D images.

### Human brainstem

**Figure 4:**
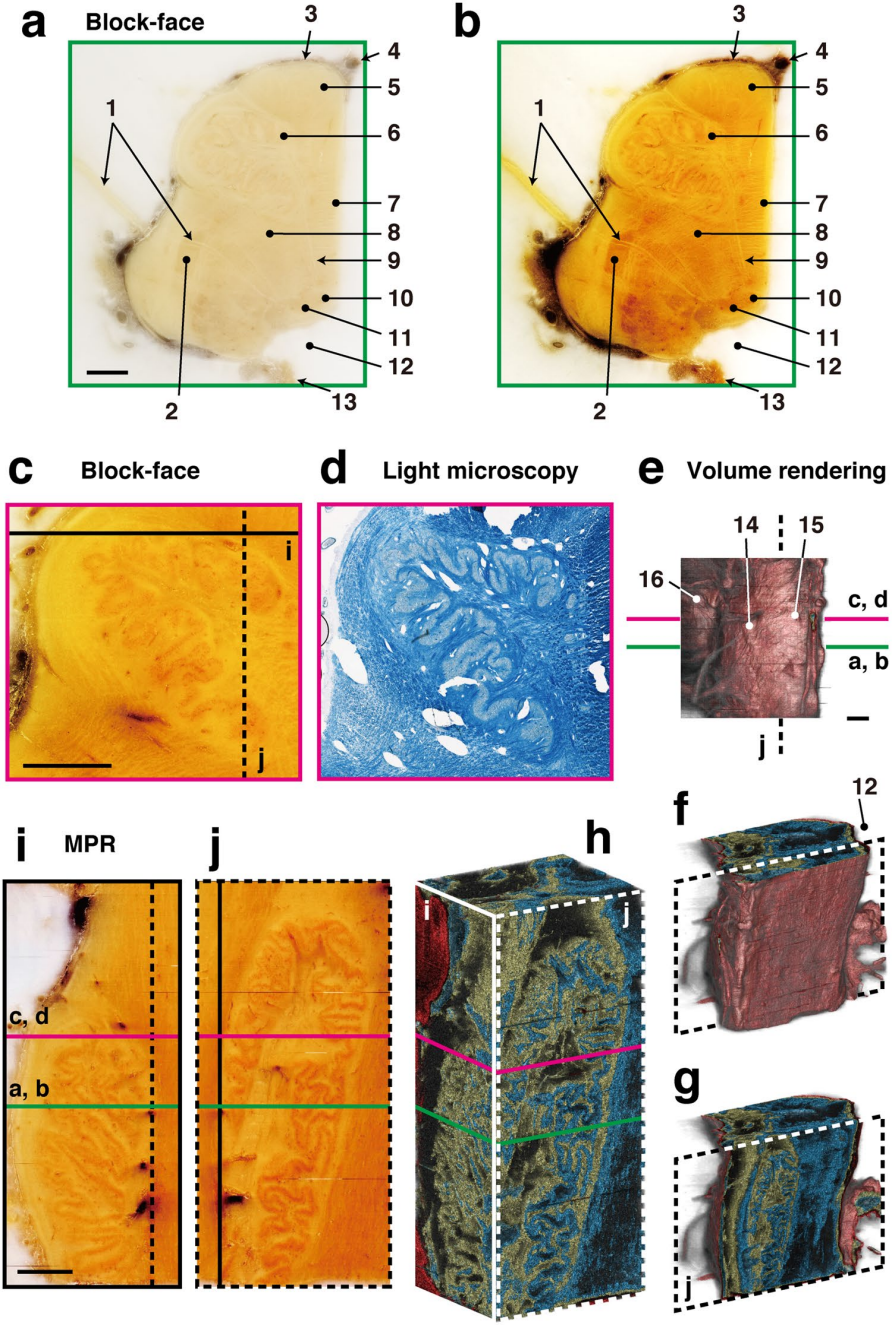
Correlative 2D and 3D images of human brainstem. The right half of the brainstem was cut into sections at a thickness of 20 µm. Volume rendering and MPR images were reconstructed from 1275 block-face images. (**a**) A block-face image taken in the horizontal plane is shown as observed by the naked eye. (**b**) The image was processed to enhance the contrast between nerve nuclei and fibers. (**c**,**d**) A block-face image was correlated with a section stained with LFB to confirm the nerve nuclei and fibers. (**e**–**h**) Volume rendering shows the surface of the brainstem (**e**,**f**), and the folds in the olivary nucleus (**g**,**h**). (**i**,**j**) MPR images show real-color images of the olivary nucleus in the coronal plane (**i**), and the sagittal plane (**j**). 1: vagus nerve rootlets, 2: spinal nucleus of trigeminal nerve, 3: pia matter, 4: basilar artery, 5: pyramidal tract, 6: principal olivary nucleus, 7: medial lemniscus, 8: reticular formation, 9: hypoglossal nerve rootlets, 10: hypoglossal nucleus, 11: dorsal vagal nucleus, 12: fourth ventricle, 13: choroid plexus, 14: olive, 15: pyramid, 16: pons. Bars: 2 mm.

The brainstem can be affected by a number of neuronal diseases, such as schwannoma, glioma, angioma, and microvascular compression, and is frequently the target of radiosurgery and craniotomy. The CoMBI system can provide details regarding the fine 3D microanatomy of the brainstem, which is essential for successful surgery (Fig. 4). The block-face of formalin-fixed brainstem tissue has low contrast on naked eye observation (Fig. 4a). As the block-face images produced by the CoMBI system are color digital images, they can be processed to enhance the contrast and thus allow identification of neuronal nuclei and roots as brown and yellow, respectively (Fig. 4b). For confirmation, frozen sections were collected from the positions indicated in MPR and volume rendering images (line d in Fig. 4e,h–j), and stained with Luxol fast blue (LFB), which colors nerve roots dark blue. The correlation between the block-face images and micrographs of LFB-stained sections confirmed the identity of nerve nuclei and roots in both sets of images (Fig. 4c and d). 3D reconstruction was performed using serial block-face images. Volume rendering showed both the surface of the brainstem (Fig. 4e) and the internal structures (Fig. 4f–h). The expanse of the olivary nuclei was presented in 3D by volume rendering (Fig. 4h) and MPR (Fig. 4i and j). This ability to obtain 3D microanatomy is useful not only for surgeons but also for medical students when studying neuroanatomy using textbooks based only on the histology of 2D sections.

### Stag beetle larvae

**Figure 5:**
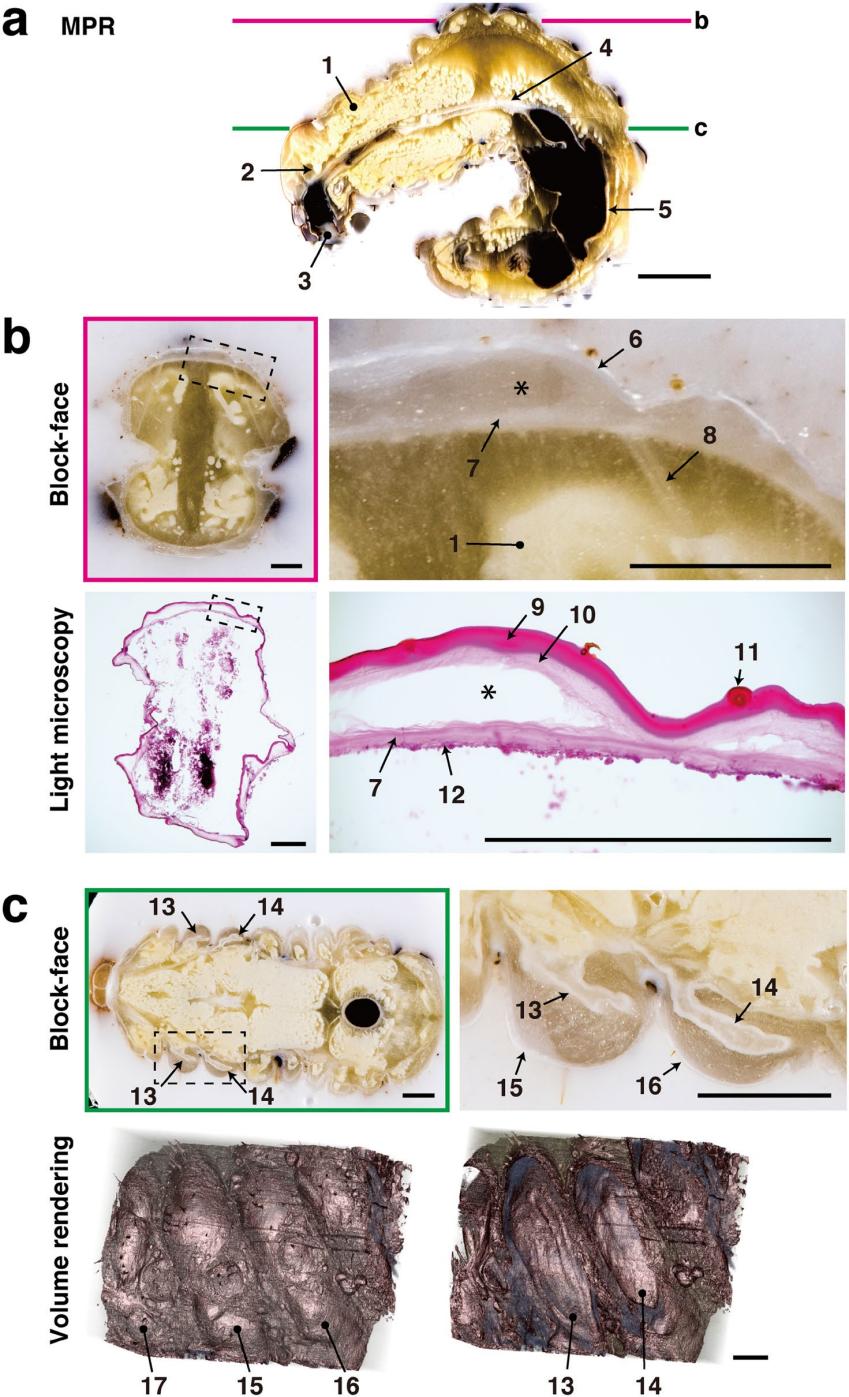
Correlative 2D and 3D images of a stag beetle larva. A whole stag beetle larva was cut into sections at a thickness of 20 µm. MPR and volume rendering were reconstructed from 1064 block-face images. (**a**) The sagittal plane was reconstructed by MPR. (**b**) Sections were stained with H&E, and correlated with block-face images. The gap between the newly developing pupal cuticle and the old larval cuticle was maintained in both the block-face image and the section (asterisk). (**c**) Two pairs of wings are shown in the block-face image. Volume rendering shows the surface structures of the thorax, and a forewing and hindwing within the thorax. 1: fat body, 2: brain, 3: oral cavity, 4: midgut, 5: hindgut, 6: larval cuticle, 7: pupal cuticle, 8: muscle, 9: larval exocuticle, 10: larval endocuticle, 11: hair, 12: epidermis, 13: forewing, 14: hindwing, 15: mesothorax, 16: metathorax, 17: prothorax. Bars in a: 5 mm, b, c: 1 mm.

Insects show a remarkable degree of morphological diversity, and are commonly used for studies of morphogenesis. The prepupal stage of holometabolous insects is suitable for analyzing newly developing structures beneath the old larval cuticle. However, such analyses are difficult at this stage by dissection, because most internal tissues of the prepupae are fragile due to disintegration. Stag beetle larvae (prepupae) were examined using the CoMBI system (Fig. 5), and the reconstructed images by MPR revealed the detailed internal structures (Fig. 5a). Sections were obtained from the position indicated in the MPR images (line b in Fig. 5a), and stained with H&E to observe the tissues by light microscopy (Fig. 5b). The correlations between microscopic and block-face images indicated that the gap between the larval cuticle and the newly developing pupal cuticle can be observed under intact conditions. The developing wings were observed in both block-face images and 3D images generated by volume rendering (Fig. 5c). The developing wings were clearly represented in 3D in the intact position within the mesothorax and metathorax.

## Discussion

Previously, the correlation between 2D and 3D data was achieved for specimens smaller than a millimeter by scanning electron microscopy (SEM) combined with an automatic tape-collecting ultra-microtome (ATUM)^8^. In this method, serial ultrathin sections are collected on tape and imaged by SEM to obtain serial images for 3D reconstruction. The ultrathin sections collected by ATUM can be analyzed further using SEM followed by labeling of molecules of interest. Our CoMBI system allows the analysis of larger specimens embedded in frozen blocks, while SEM with ATUM require specimens to be fixed and embedded in resin. Frozen blocks can be made from specimens in size from several millimeters to several centimeters, and in various conditions, e.g., fresh-frozen or fixed using various fixatives. Frozen sections are compatible with various histochemical or immunofluorescence staining procedures for obtaining various information regarding anatomy and molecules of interest. Actually, we used CoMBI to obtain correlative 2D and 3D data of specimens varying in species, size, and fixation conditions (Figs 2–5). Thus, our CoMBI system expands the field of correlative 2D and 3D imaging by covering relatively large specimens, and most subjects commonly analyzed in biological laboratories by conventional light microscopy.

The demand for both 3D anatomical and 3D molecular imaging are increasing in various research fields, such as phenomics of knockout mice^9^, connectome of whole brain^10^, and tissue engineering^11, 12^. A number of modalities and methods are recently available for 3D imaging, and each has its own range of applications, and provides information about anatomy and/or molecules of interest^11^. As an optical based 3D imaging methods, there is a classical method for obtaining 3D volume data using serial sections; sections are collected thoroughly, colored with a certain staining, and then imaged using a microscope or slide scanner^13^. In this method, however, care should be taken when handling images as sections may be damaged and deformed^14^, and sections after 3D imaging cannot be used for different staining procedures. Recent optical based 3D imaging systems, such as two-photon confocal microscope, light-sheet microscope^15^, etc. can clearly visualize the signals of fluorescently labeled molecules in 3D using a clarified specimen from submillimeter to a centimeter in size^16^. Among them, light-sheet microscope has been attracting attention since it is capable to acquire high-resolution 3D images rapidly, and cost-effective^17^. However, anatomical information should be interpreted carefully, as clarified specimens may have the deformation artifact due to the shrinkage or expansion during clearing process^18^. Both X-ray and magnetic resonance based modalities, such as micro computed tomography (µCT) and magnetic resonance imaging (MRI), are becoming more readily accessible in the laboratory for non-destructive 3D imaging of specimens on the scale of several centimeters^19–21^. µCT can be used to generate grayscale 3D images with a voxel size from sub-micrometers to several micrometers^22^. µCT is suitable for visualization of bones, and soft tissues with successful use of contrast enhancing reagents by circulation or infiltration^23^. By µCT, most developing organs of mouse embryos could be clearly visualized on E10.5^24^, E12.5^9, 19^ and E15.5^20^ using contrast reagents. MRI is commonly used to observe neuronal tissue, although still not as familiar as µCT in the laboratory. MRI could visualize internal structures of larger specimens, such as the human^25–27^, cat^28^ or mouse brainstem^29^. Our CoMBI system was developed utilizing block-face imaging to avoid deformation artifacts, which are seen in other optical based methods, so that it obtains the intact 3D anatomical data of the specimens. Using 3D volume data obtained by the CoMBI system, most of the developing organs of mouse embryos on E10.5 can be observed in natural color (Fig. 2), and also in enhanced color stained with reagents, such as hematoxylin (Fig. 3). The CoMBI can also visualize post-mortem human brain and stag beetle larvae, of which contrast reagents hardly circulate or infiltrate (Figs 4 and 5). Thus, the CoMBI system can obtain 3D volume data of a wide variety of biological specimens in various condition, although it is performed in the destructive manner. To obtain both 3D anatomical and molecular information from a single specimen, multimodal methods, e.g., single photon emission computed tomography (SPECT)/CT^30^ and positron emission tomography (PET)/MRI^31^, and new biomarkers and contrast reagents have been developed^32, 33^. Combination of multimodal methods and use of biomarkers complements the weak points of each imaging modality, and widens the applicability^34^. The CoMBI method also obtains both anatomical and molecular information, although the molecular information is 2D. Our method has different range of applicability from other multimodal imaging methods, and can be one of alternative imaging methods for obtaining multiple information from single specimens.

The 3D imaging may lead a researcher to consider computer environment. We performed image acquisition, storage, and processing using personal computers (see Methods for specifications). Image processing from RAW-JPEG conversion to DICOM data takes several hours after the acquisition of serial block-face images. Therefore, it is possible to obtain 3D data from multiple specimens within 1 day. In fact, we routinely use the CoMBI system to examine the causes of death due to gene modifications, and can determine 3D anatomy from three to four newborn mice per day (unpublished). RAW-JPEG conversion and image registration represent the heaviest processing loads and require large amounts of memory. Therefore, we recommend 16 GB or more of memory for this technique. The data volume of an original block-face image series can vary from 7 to 34 GB per specimen depending on the image size, number, and format (Table 1). The original RAW image series is used to produce a JPEG image series and a DICOM image series, which are then used for image registration and 3D reconstruction, respectively. These derivative image series did not exceed 1 GB. Image processing was performed using Adobe Photoshop and ImageJ, which are commercially available and open source software, respectively, and are likely to be familiar to most researchers. Many open-source programs are available for 3D reconstruction^35^, and researchers can select any software that is suitable for their computer environment; we used OsiriX in this study, as it works well in our computer environment^36^.

The CoMBI system described here consists of a combination of self-made devices, including the controller, sensor, illumination, and cleaning brush, which cost only US$124 (Supplementary Table S1), and ready-made devices, including a cryostat, DSLR camera and accessories, fluorescent microscope and personal computer. Among the ready-made devices, the DSLR camera and its accessories may be unfamiliar as biological research tools, and cost about US$4922 (Supplementary Table S1). The self-made devices are designed such that they can be easily attached to the cryostat and removed after use to allow the cryostat to be used by other researchers. Thus, if a cryostat, fluorescent microscope and personal computer are already available as laboratory’s own or common facility, the CoMBI system would cost US$5046 to assemble. Construction of the whole CoMBI system, including a cryostat, fluorescent microscope and personal computer, would cost approximately US$115250 (Supplementary Table S1), which is more expensive than self-made light sheet microscope (US$57000; OpenSPIM^37^), and less expensive than commercial light sheet microscope (US$260000), µCT (US$200000–1 million), MRI ($1 million per Tesla of magnetic field), and SEM for ATUM specimens (US$200000–600000). Our CoMBI system can be one of cost effective 3D imaging system, which has different properties from other modalities.

CoMBI represents the first method reported to date for correlating 3D anatomy and various 2D microscopic images of biological specimens on a scale ranging from several millimeters to several centimeters. This technique can be applied to a wide variety of specimens, as demonstrated in this study using mouse embryos, human brainstem samples, and insect larvae. We have presented all information, including parts, codes, and drawings, required to reproduce and use the CoMBI system around the world. We believe that the CoMBI method will prompt many researchers to join the field of 3D imaging, and improve the quality and reliability of 2D/3D morphological analyses.

## Methods

### Cryostat

A Leica CM3050S cryostat (Leica Microsystems K.K., Tokyo, Japan) with a disposable knife (Leica 818) was used for sectioning. A small piece of light-absorbing sheet (approximately 8 × 15 mm, Spectral Black; Acktar Japan, Tokyo, Japan) was attached to the handle to absorb light from the photointerrupter and thus convey the handle/block position as described below.

### Camera

The block-faces were photographed with a DSLR camera (Nikon D810; Nikon, Tokyo, Japan) with a macro lens (Tamron AF180 mm F/3.5 Di LD [IF] MACRO1:1; Tamron, Saitama, Japan). The camera was powered by an AC adapter (Nikon EH-5b) and set on a tripod (Husky #1003; Toyo Trading, Kyoto, Japan) with a geared head (Manfrotto 410; Manfrotto Distribution KK, Tokyo, Japan). The camera shutter release was triggered by connecting three pins: release pin, focus pin, and ground pin of a remote code (Nikon MC-22A). Images were saved on the SD card or transferred onto a computer using a USB3 cable. The camera was placed in front of the block and kept at room temperature outside the cryostat chamber. The camera system can take a block-face area of 34.5 × 23.0 mm as a single image of 7360 × 4912 pixels at the minimal working distance of 25 cm. Thus, the minimal pixel size of the image was calculated as 4.68 × 4.68 µm/pixel.

### Self-made devices

The self-made devices, including the controller, sensor, cleaner, and illumination, were assembled in our laboratory (Supplementary Figs S1–S4). All components and materials used in these devices are listed in Supplementary Table S1. Parts were purchased from various parts stores (RS Components, Yokohama, Japan; Akizuki Denshi, Tokyo, Japan; Sengoku Densho, Tokyo, Japan; Marutsu Elec, Tokyo, Japan). The enclosure and frames for the devices were made of acrylic plates, 2 mm thick, which were processed with a laser cutter (Smart Laser CO_2_; smartDIYs, Yamanashi, Japan). The drawings were made using the open source software, Inkscape v0.91 (https://inkscape.org/), and saved as scalable vector graphics (svg) files with a resolution of 90 dpi (Supplementary Fig. S6).

### Controller

The controller connects the camera and the sensor, cleaner, and illumination (Fig. 1, Supplementary Figs S1, S2, and Note). The electronic components used were microcontrollers (ATtiny85), an optocoupler, resistors, capacitors, a DC/DC converter, and LED with a bracket. The controller contains two microcontrollers—one is connected to the sensor for detection of the handle/block position, optocoupler for triggering the camera shutter release, and LED to visually confirm the shutter release (Fig. 1a and c, Supplementary Figs S1, S2, and Video S1), and the other is connected to the cleaner to regulate brush movement (Fig. 1e, Supplementary Figs S1, S4, and Video S4).

### Illumination

The samples were illuminated with two LED lamps (3.8 W) located in the cryostat chamber, which illuminated the block-face evenly and diagonally (Fig. 1, Supplementary Figs S1 and S4). The illumination was connected to the controller using a DC plug.

### Sensor

A reflective photosensor was attached 6 mm apart from the surface of the cryostat handle (Fig. 1, Supplementary Figs S1, S3, and Video S1). The sensor detects reflected light from the surface of the cryostat handle. The brightness of reflected light is converted to a 10-bit analog value, and sent to the microcontroller (Supplementary Note). The values of the cryostat handle and the light absorbing sheet are around 60 and 900, respectively. The sensor was connected to the controller using a 4-pin stereo plug.

### Cleaning brush

The brushes were attached behind the knife holder to keep the block-face clean (Supplementary Figs S1, S4, and Video S4). The brush movement is generated by a servomotor under the control of a microcontroller (Supplementary Note). The cleaner was connected to the controller using a 3-pin stereo plug.

### Program

The codes for releasing the shutter and regulating the cleaning brushes were written using Arduino Software (freeware, http://www.arduino.cc/; Arduino SRL, Scarmagno, TO, Italy) (see Supplementary Note). An Arduino UNO R3 microcontroller board (Arduino SRL) was used to upload the codes to the ATtiny85 microcontrollers according to the tutorials (http://highlowtech.org/?p=1695; MIT Media Lab., Cambridge, MA). The code for releasing the shutter configures the photoreflector, optocoupler, and LED. When the photoreflector detects the light-absorbing sheet on the cryostat handle, the optocoupler triggers the shutter release by connecting the release pin and the focus/ground pins. The LED visually indicates shutter release. The code for regulating the cleaning brush configures a servomotor to repeatedly move the brush.

### Frozen blocks

Frozen blocks were prepared as a right circular cylinder 14 or 26 mm in diameter (Supplementary Fig. S5). The maximum height of the block is 25 mm depending on the specimen feed capacity of the cryostat. A black rubber ring made from a chloroprene rubber sponge sheet 1 mm thick (Chiyoda Rubber, Tokyo, Japan) was inserted at the bottom of the block (Supplementary Figs S5 and S6).

### Biological specimens

Pregnant Slc:ICR mice were purchased from SLC (Hamamatsu, Shizuoka, Japan). Mice were killed by cervical dislocation under isoflurane anesthesia (Wako Pure Chemical Industries, Osaka, Japan). Animal experiments were approved by the Animal Care and Experimentation Committee, Gunma University (#15-060). Human cadavers were donated to Gunma University for their use in both research and education with the written informed consents from the donor and the next of kin, and fixed with 3.7% formaldehyde (Nippon Kasei Chemical, Tokyo, Japan) by injection through the left radial artery. The brain was removed and stored in 3.7% formaldehyde at room temperature until use. The brainstems of the human cadavers were used with approval from the Ethics Committee of Gunma University School of Medicine (#14-15). Stag beetles (*Cyclommatus metallifer*) were maintained in the laboratory as described previously^38, 39^. Larvae were chilled on ice before use. Mouse embryos, human brainstem specimens, and stag beetle larvae were mounted in OCT compound (Sakura Finetek, Tokyo, Japan), and quickly frozen using liquid nitrogen and 2-methylbutane (Wako Pure Chemical Industries). All methods were performed in accordance with the relevant guidelines and regulations.

### Cryostat setting

The chamber temperature was set at –15 °C, and section were cut at a thickness of 10 or 20 µm. To set the auto-drive mode of the cryostat, the “Sectioning Window” was set above the knife. While the block is moving slowly in this window, the block-face was photographed when the sensor detected the light-absorbing sheet on the handle. The speed of the “Sectioning Window” was set to “25%,” which was sufficiently slow to avoid motion blur. Actual sectioning was carried out just after photography in the “Returning Window.” An anti-roll plate is necessary for both block-face imaging and collecting sections. The anti-roll plate prevents scattering of sections as debris and keeps the block-face clean during imaging. It also works in the regular way to keep the sections flat before collection on glass slides. Self-made covers are used to stabilize the chamber temperature and avoid fogging (Supplementary Figs S1 and S4).

### Camera setting

The shutter speed was set to 1/250 s, which was sufficiently fast to photograph a moving block without motion blur. The aperture size was set to f/8, which was small enough to achieve sufficient depth of focus. The sensitivity was set to ISO 200, which was sufficiently low to reduce noise. The camera was carefully set perpendicular to the block-face.

### Procedure for acquiring both block-face image series and frozen sections

1. Check the camera is positioned perpendicular to the block-face at the position of shutter release.
2. Place a scale on the block-face to acquire the calibration image. Take one image manually, and then remove the scale.
3. Start auto-drive mode of the cryostat, and take serial images of the block-faces automatically (Supplementary Video S1).
4. Pause arbitrary axial position, and collect sections on the glass slide as usual (Supplementary Video S3).
5. Restart auto-drive mode of the cryostat to continue block-face imaging.

### Image processing

Images were saved in the RAW or JPEG format and processed using Adobe Bridge, Adobe Photoshop CC (San Jose, CA) and ImageJ v1.47 64-bit (NIH, Bethesda, MA) on an iMac Late 2012 model (CPU: 3.4 GHz Intel Core i7, DRAM: 32GB 1600 MHz DDR3, graphics: NVIDIA GeForce GTX 680MX 2GB, and storage: 3TB HDD) or a MacBookPro Early 2015 model (CPU: 3.1 GHz Intel Core i7, DRAM: 16GB 1866 MHz DDR3, graphics: Intel Iris Graphics 6100, and storage: 512GB SSD) (Apple Japan, Tokyo, Japan). OsiriX v6.5 64-bit (a DICOM viewer; Pixmeo, Bernex, Switzerland) was used to convert JPEG to DICOM format with the JPEG to DICOM plug-in, and to reconstruct MPR and volume rendering images^40^.

### Procedure for processing image series

1. Convert RAW images into JPEG images. At the same time, crop images to remove the unnecessary area and resize images as 5, 10, or 20 µm/pixel in accordance with the calibration image (Supplementary Fig. S7). Do not remove the outer circumference of the block in this cropping process, as it is necessary for subsequent image registration. Cropping and resizing reduce the data volume and processing load for image registration.
2. Register image series using the ImageJ plug-in “Image Stabilizer” (http://www.cs.cmu.edu/~kangli/code/Image_Stabilizer.html)(Supplementary Fig. S7 and Video S5), and save as a new image series. Note that the pre-installed plug-in “StackReg” also works well, however, very occasionally, it may distort whole shape of the specimen.
3. For 3D reconstruction, create a color image series and grayscale image series. Execute the following commands automatically using Photoshop “Actions” and “Batch” processing: crop images to remove unnecessary areas and show only the specimen, adjust brightness, save as new image, convert to grayscale, invert brightness, and save as new image (Supplementary Fig. S7).
4. Import the image series (step 3) into OsiriX using the JPEG to DICOM plug-in. Use color image series for “3D-MPR”, and the grayscale image series for “3D Volume Rendering”. The XYZ voxel sizes are referred to step 1 (resize) and the thickness of section.

### Histochemical and immunofluorescence staining

Luxol fast blue (LFB) staining was performed according to the manufacturer’s protocol (LFB Solution; Muto Pure Chemicals, Tokyo, Japan). H&E staining was performed using hematoxylin solution (Muto Pure Chemicals) and 1% eosin in water (Wako Pure Chemicals). Immunofluorescence staining was performed using a Mouse-on-Mouse immunodetection kit (Vector Laboratories, Burlingame CA), anti-VAMP2 antibody (1:200, cl:69.1; Synaptic Systems, #104211, Goettingen, Germany), anti-cyclin D1 antibody (1:50, cl:SP4; Thermo Fisher Scientific KK, #RM-9104-SO, Yokohama, Japan), AlexaFluor488-conjugated donkey anti-rabbit IgG F(ab’)_2_ fragment (1:500; Jackson ImmunoResearch, West Grove, PA), and Rhodamine RedX-conjugated donkey anti-mouse IgG F(ab’)_2_ fragments (1:500, Jackson ImmunoResearch). Microscopic images were obtained using a Nikon AZ-100 (Tokyo, Japan) and a cooled CCD camera (SPOT RT3; Diagnostic Instruments, Sterling Heights, MI).

## Electronic supplementary material

Supplementary Information

Supplementary Video S1

Supplementary Video S2

Supplementary Video S3

Supplementary Video S4

Supplementary Video S5
