# Supplementary material for: A novel imaging method for correlating 2D light microscopic data and 3D volume data based on block-face imaging
Source: Sci Rep. 2017 Jun 16;7:3645. doi: 10.1038/s41598-017-03900-9 (PMC5473845; doi:10.1038/s41598-017-03900-9)
Supplement: Supplementary file 1 — Supplementary Information [file 41598_2017_3900_MOESM1_ESM.pdf]

A novel imaging method for correlating 2D light microscopic data and 3D volume data based on block-face imaging

Yuki Tajika*, Tohru Murakami, Keiya Iijima, Hiroki Gotoh, Maiko Takahashi-Ikezawa, Hitoshi Ueno, Yuhei Yoshimoto, Hiroshi Yorifuji

PDF file

Supplementary Table S1 | List of components used in the CoMBI system and approximate costs.
Supplementary Figure S1 | Installation of the CoMBI system
Supplementary Figure S2 | Controller
Supplementary Figure S3 | Sensor
Supplementary Figure S4 | Illumination, cleaning brush, and covers
Supplementary Figure S5 | Procedure for making the frozen blocks with a right cylindrical shape and black background
Supplementary Figure S6 | Drawings for self-made devices
Supplementary Figure S7 | Procedure for image processing
Supplementary Note | Codes for releasing the camera shutter and regulating the cleaning brush

MOV files

Supplementary Video S1 | Imaging the block-face serially
Supplementary Video S2 | 3D reconstruction from the serial block-face images
Supplementary Video S3 | Collecting a section while pausing the block-face imaging
Supplementary Video S4 | Cleaning the block throughout imaging
Supplementary Video S5 | Image registration

Supplementary Table S1 | List of components used in the CoMBI system and approximate costs.

Components of self-made devices

Categories	Parts	Part#	Manufacturers	Vendors	Unit prices JPY	Quantities	Amounts JPY	Amounts US$	Sub total US$
Essential electronic components	Microcontroller	ATtiny85	Atmel, San Jose, CA, USA	Marutsu	215	2	430	$4.22	
	Photointerrupter	RPR-220	Rohm, Kyoto, Japan	Sengoku	137	1	137	$1.34	
	Optocoupler	TLP785(GB)	Toshiba, Minato, Tokyo, Japan	Akizuki	20	1	20	$0.20	
	LED with bracket	DB9TCHR	Sato Parts, Shibuya, Tokyo, Japan	Marutsu	140	1	140	$1.37	
	DC-DC converter	R-78E5.0-0.5	RECOM, Gmunden, Austria	Akizuki	350	1	350	$3.43	
	Stacked ceramic capacitor	10μF-25V	Murata Manufacturing, Nagaokakyo, Kyoto, Japan	Akizuki	30	2	60	$0.59	
	Resistor 220, 10k		(No brand)	RS Components	1	4	4	$0.04	
	Universal board	PU45x64	Daisen Electronics Industrial, Osaka, Japan	Sengoku	242	1	242	$2.37	
	Cheek brush	Haruhime	Daiso, Higashi-Hiroshima, Hiroshima, Japan	Daiso	100	1	100	$0.98	
	Micro servo motor	SG92R	Tower Pro, Taiwan	Akizuki	500	1	500	$4.90	
	LED lamp	OSLA03X3W00	OptoSupply, Fo Tan, N.T., Hong Kong	Akizuki	900	2	1800	$17.65	
	Switching AC adoptor 12V-1A	NP12-1S1210	Go Forward Enterprise, Taichung, Taiwan	Akizuki	750	1	750	$7.35	
Panel mount components	DC jack 2.1 mm	MJ-21	Marushin Electric, Kawasaki, Kanagawa, Japan	Marutsu	200	2	400	$3.92	
	DC plug 2.1 mm	MP121C	Marushin Electric, Kawasaki, Kanagawa, Japan	Akizuki	60	1	60	$0.59	
	3.5 mm stereo plug, 4-pins	435_IT	Comon, Saitama, Saitama, Japan	Sengoku	125	1	125	$1.23	
	3.5 mm stereo panel mount jack, 4-pins	MJ-079	Marushin Electric, Kawasaki, Kanagawa, Japan	Sengoku	163	1	163	$1.60	
	2.5 mm stereo plug, 3-pins	MP-025MH	Marushin Electric, Kawasaki, Kanagawa, Japan	Sengoku	115	1	115	$1.13	
	2.5 mm stereo panel mount jack, 3-pins	MJ-070	Marushin Electric, Kawasaki, Kanagawa, Japan	Sengoku	105	1	105	$1.03	
	Toggle switch, maintained	MS500AB	Miyama Electric, Ohta, Tokyo, Japan	Marutsu	204	4	816	$8.00	
	Toggle switch, momentary	MS-500B-B	Miyama Electric, Ohta, Tokyo, Japan	Marutsu	179	1	179	$1.75	
	Binding post	MB-126G	Avvicon, Tainan, Taiwan	Akizuki	150	3	450	$4.41	
	Rocker switch	Fujisoku SLE210K-9	Nidec Copal Electronics, Shinjuku, Tokyo, Japan	Marutsu	180	1	180	$1.76	
Wiring components	IC socket 6P		(No brand)	Marutsu	7.4	2	14.8	$0.15	
	IC socket 8P		(No brand)	Akizuki	10	1	10	$0.10	
	XH connector contact	SXH-001T-0.6	J.S.T.MFG. Osaka, Japan	Marutsu	10	26	260	$2.55	
	XH connector housing 2P	XHP-2	J.S.T.MFG. Osaka, Japan	Marutsu	22	9	198	$1.94	
	XH connector housing 3P	XHP-3	J.S.T.MFG. Osaka, Japan	Marutsu	26	2	52	$0.51	
	XH connector housing 4P	XHP-4	J.S.T.MFG. Osaka, Japan	Marutsu	30	1	30	$0.29	
	XH connector shrouded header, top entry, 2P	B2B-XH-A	J.S.T.MFG. Osaka, Japan	Marutsu	23	9	207	$2.03	
	XH connector shrouded header, top entry, 3P	B3B-XH-A	J.S.T.MFG. Osaka, Japan	Marutsu	24	2	48	$0.47	
	XH connector shrouded header, top entry, 4P	B4B-XH-A	J.S.T.MFG. Osaka, Japan	Marutsu	32	1	32	$0.31	
	Slim robot cable 2P, 3m	KRT AWG28x2C	Kyowa Harmonet, Fushimi, Kyoto, Japan	Sengoku	330	0.2	66	$0.65	
	Slim robot cable 3P, 3m	KRT AWG28x3C	Kyowa Harmonet, Fushimi, Kyoto, Japan	Sengoku	420	0.2	84	$0.82	
	Slim robot cable 4P, 3m	KRT AWG28x4C	Kyowa Harmonet, Fushimi, Kyoto, Japan	Sengoku	490	0.2	98	$0.96	
	polyvinyl chloride wire, 0.2SQ, 10 m	AVRB0.2SQ Black/Red	(No brand)	Marutsu	280	0.05	14	$0.14	
	Tin coating copper wire	TCW 0.6mm 10m	Kyowa Harmonet, Fushimi, Kyoto, Japan	Akizuki	210	0.01	2.1	$0.02	
Plastic components	M3 screw, 10 mm	M3X10polybis	Neji-no-Takayama, Katsushika, Tokyo, Japan	Marutsu	1.2	33	39.6	$0.39	
	M3 nut	M3polynut	Neji-no-Takayama, Katsushika, Tokyo, Japan	Marutsu	16	16	256	$2.51	
	M3 screw, countersunk head, 12 mm	GB-PFHS-M312-100P	Linkman, Fukui, Fukui, Japan	Marutsu	3.8	6	22.8	$0.22	
	Spacer with M3 female thread, 30 mm	AS-330B	Hirosugi-Keiki, Kawasaki, Kanagawa, Japan	Sengoku	53	8	424	$4.16	
	Spacer with M3 female thread, 12 mm	DS312	Takachi Electronics Enclosure, Kawaguchi, Saitama, Japan	Marutsu	15	2	30	$0.29	
	Spacer, M3, 3mm	C-303	Hirosugi-Keiki, Kawasaki, Kanagawa, Japan	Marutsu	15	8	120	$1.18	
	Acrylic plate, black, 320*550*2.0mm	EX502S2	Acrysunday, Shinjuku, Tokyo, Japan	Marutsu	1309	0.2	261.8	$2.57	
	Acrylic plate, clear, 320*550*2.0mm	EX001S2	Acrysunday, Shinjuku, Tokyo, Japan	Marutsu	1182	1	1182	$11.59	
	Acrylic plate, milky white translucent, 320*550*2.0mm	EX432S2	Acrysunday, Shinjuku, Tokyo, Japan	Marutsu	1241	1	1241	$12.17	
Others	Neodymium magnet with countersunk screw hole, 10*3 mm, rounded		(No brand)	Sengoku	189	4	756	$7.41	
	10*5mm clear adhere rubber feet Pad		Shenzhen Fushiyuan Rubber & Plastic Factory, Shenzhen, China	Akizuki	10	2	20	$0.20	$123.48

Commercially available devices

Categories	Parts	Part#	Manufacturers	Vendors	Unit prices JPY	Quantities	Amounts JPY	Amounts US$	Sub total US$
Sectioning	Cryostat	CM3050S	Leica Microsystems K.K., Tokyo, Japan		6162700	1	6162700	$60,418.63	$60,418.63
Block-face imaging	Digital single lens reflex camera	D810	Nikon, Tokyo, Japan		344844	1	344844	$3,380.82	
	Lens	SP AF 180mm F/3.5 Di LD [IF] MACRO 1:1	Tamron, Saitama, Japan		72144	1	72144	$707.29	
	Geared head	#410	Manfrotto Distribution K.K., Tokyo, Japan		26000	1	26000	$254.90	
	Tripod	#1033	Husky #1003, Toyo Trading, Kyoto, Japan		43600	1	43600	$427.45	
	Remote code	MC-22A	Nikon, Tokyo, Japan		3110	1	3110	$30.49	
	Power connector	EP-5B	Nikon, Tokyo, Japan		2484	1	2484	$24.35	
	AC adaptor	EH-5B	Nikon, Tokyo, Japan		9936	1	9936	$97.41	$4,922.73
Image processing	Apple iMac	27-inch, Late 2012	Apple Japan, Tokyo, Japan		252098	1	252098	$2,471.55	$2,471.55
Fluorescent imaging	Light microscope	AZ-100 (3-revolver, 3-fluorochome set)	Nikon, Tokyo, Japan		3626000	1	3626000	$35,549.02	
	Cooled CCD camera	SPOT RT3 (including software)	Diagnostic Instruments, Sterling Heights, MI, USA		1200000	1	1200000	$11,764.71	$47,313.73

Total	11755511	$115,250.11

Rate: $1=¥102 (15 Sep. 2016)

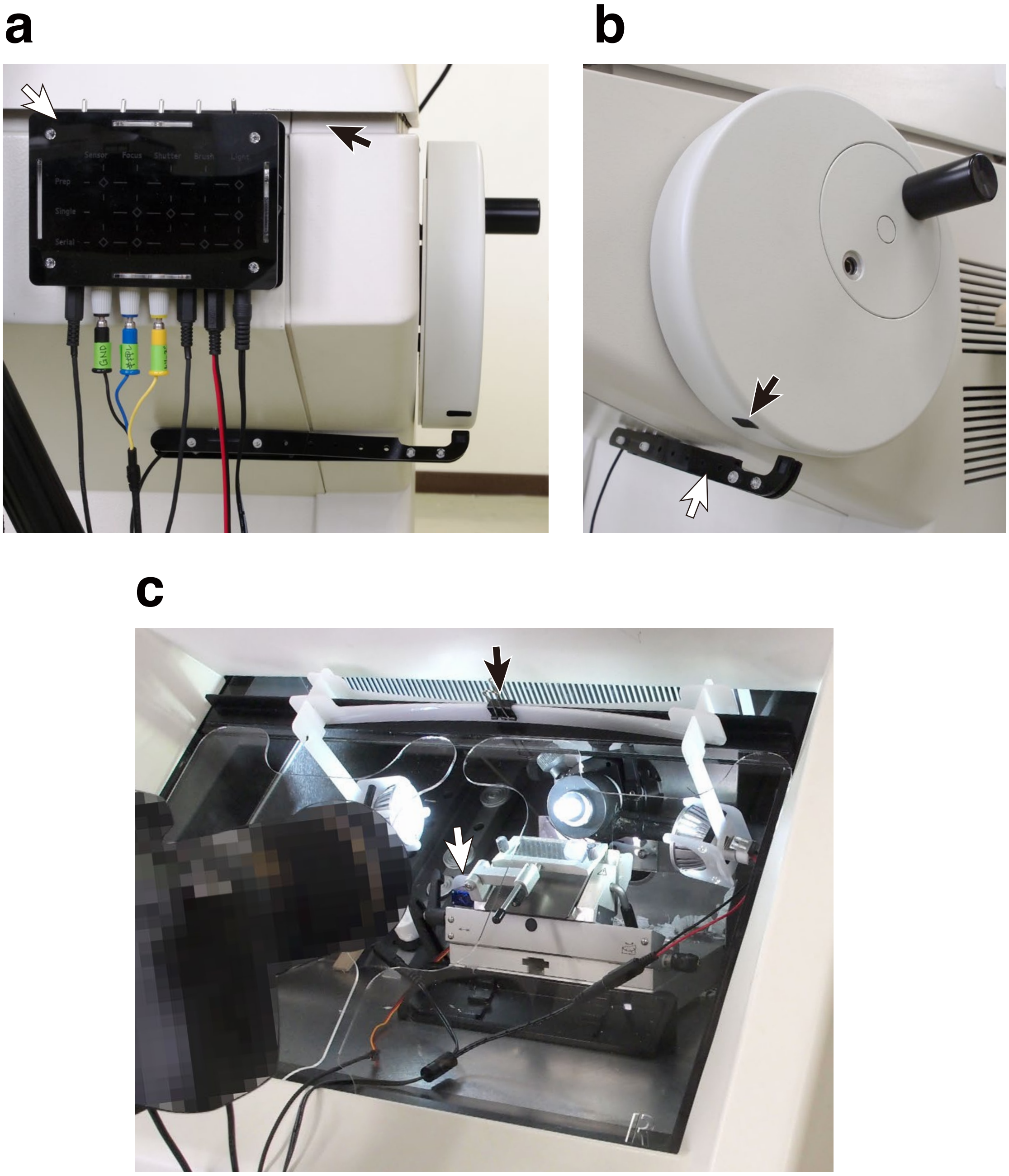

Supplementary Figure S1 | Installation of the CoMBI system. a: The controller (white arrow) is attached to the front panel of the cryostat by magnets. The plate-like projection, which is hooked to the groove (black arrow), contributes to stability of the attachment. b: The sensor (white arrow) is attached close to the handle. A light-absorbing sheet is attached to the handle (black arrow). c: The illumination is fixed using plastic plates and a clip (black arrow), and illuminates the block-face orthogonally. The cleaning brush is attached to the back of the knife holder (white arrow), and cleans the block throughout imaging. Covers are used to stabilize the chamber temperature and avoid fogging.

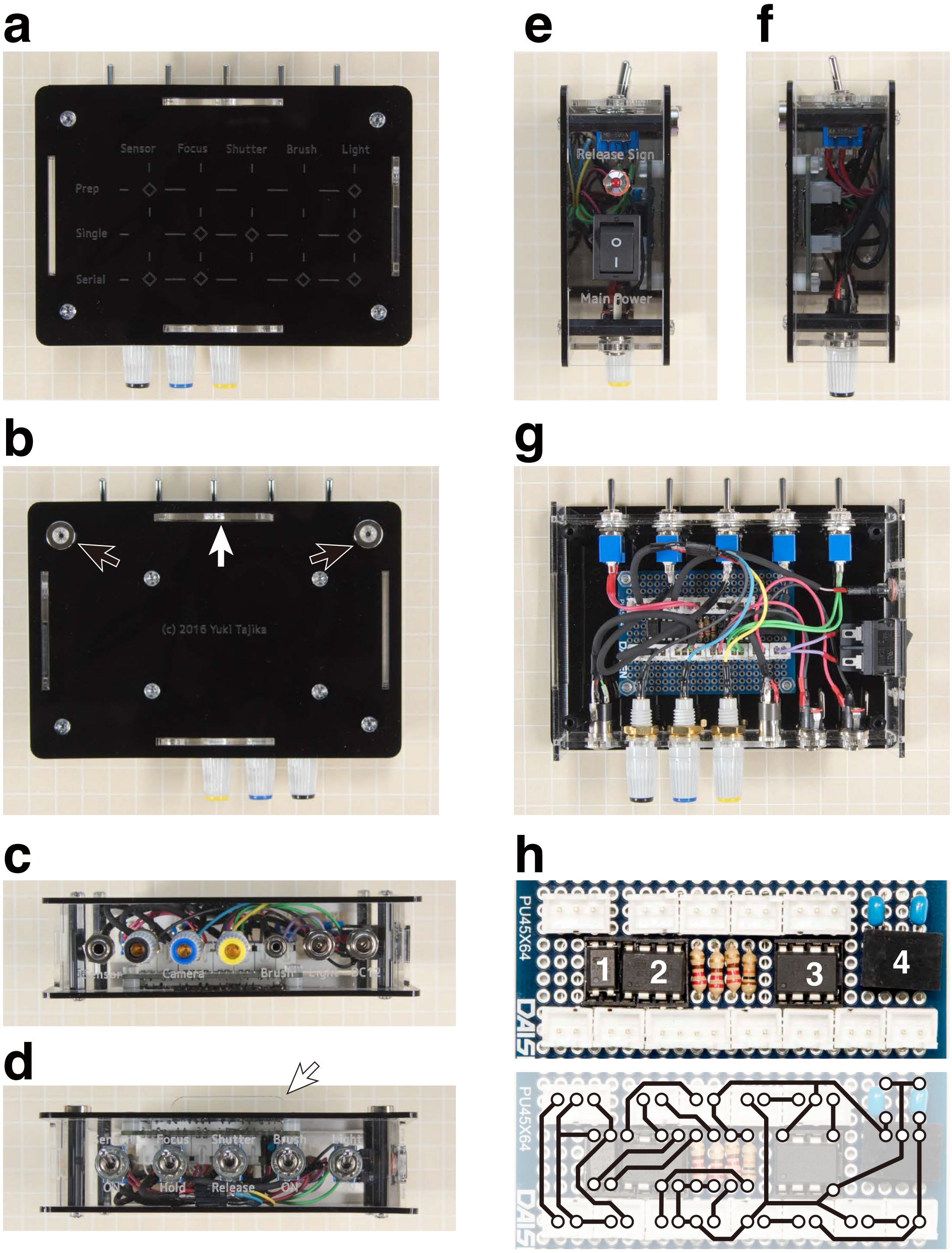

Supplementary Figure S2 | Controller. a: Schematic instructions are shown on the front panel. b: The controller is fixed to the cryostat with two magnets (black arrows in b), and the plate-like projection is hooked to the groove (white arrows in b and d). c: The jacks at the bottom connect the sensor, camera, cleaning brush, illumination, and 12 V power supply. d: Toggle switches are used to switch the sensor, brush, and illumination, and to control camera focus and shutter release. e: The LED at the right side of the panel indicates the timing of shutter release. The rocker switch is the main power switch. f: There is no function on the left side. g: The internal appearance of the controller is shown. The jacks and switches are connected to the main board. h: The main board consists of an optocoupler (1), a microcontroller for regulating the shutter release (2), a microcontroller for regulating the cleaning brush (3), a DC/DC converter for converting 12 V to 5 V (4), resistors, capacitors, and connectors, which are soldered and wired as illustrated at the bottom. Square pattern: 1 cm.

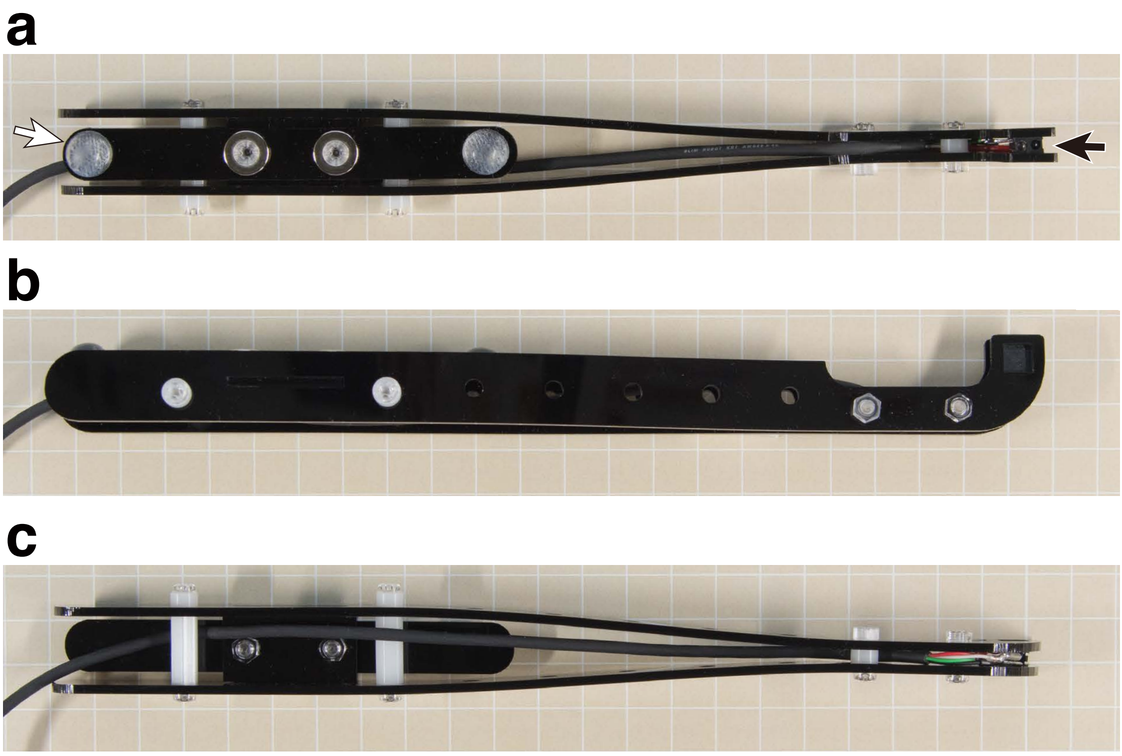

Supplementary Figure S3 | Sensor. a: The top view of the sensor is shown. A photoreflector is fixed between the plastic frames (black arrow). The sensor is attached stably to the cryostat by magnets at the central part of the plate spring, which presses silicone pads to the cryostat (white arrow). The sensor detects light reflection from the handle surface, and sends the analog value to the microcontroller. Changes in value at the light-absorbing sheet on the handle trigger camera shutter release. b, c: Side and bottom views of the sensor are shown. Square pattern: 1 cm.

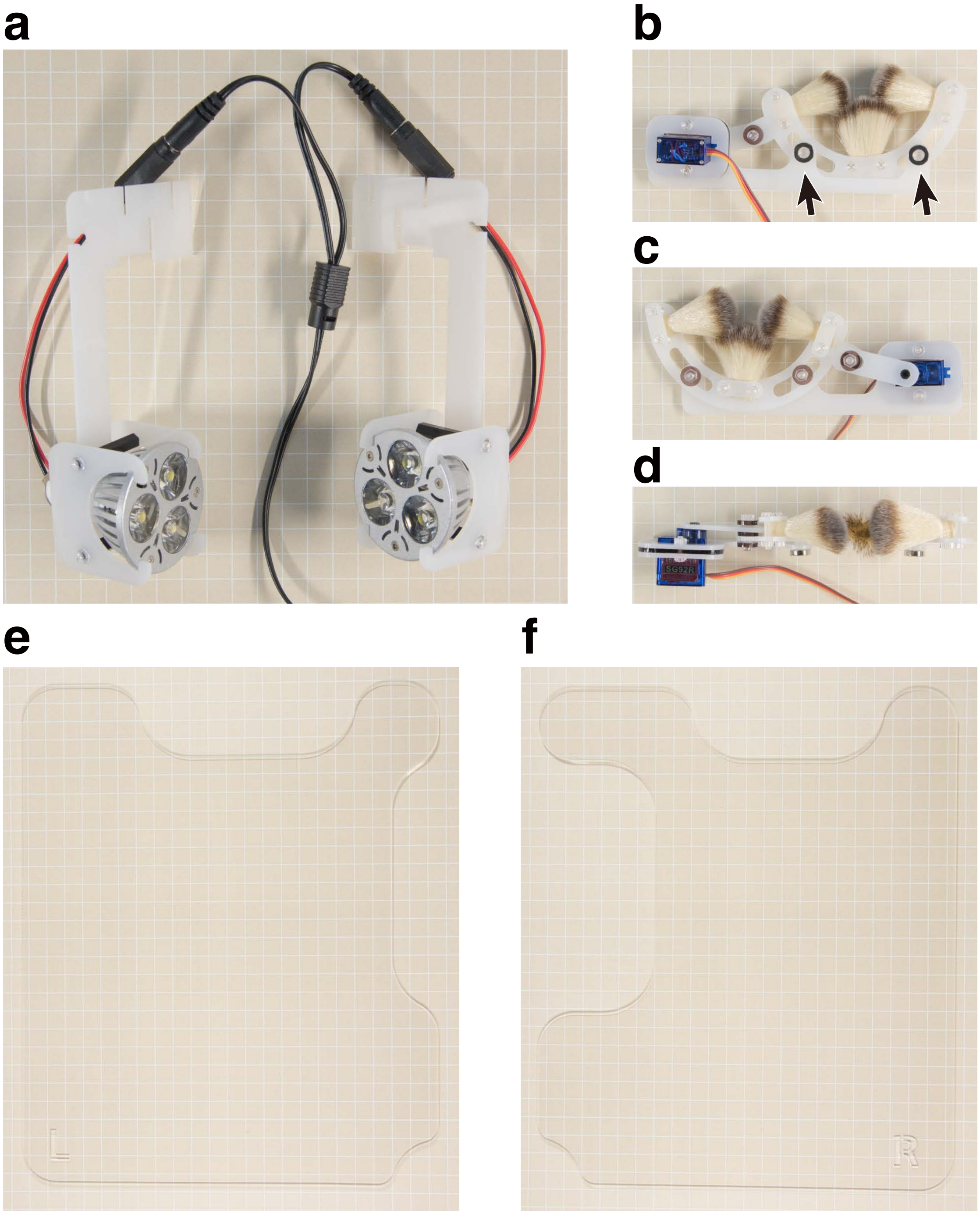

Supplementary Figure S4 | Illumination, cleaning brush, and covers. a: The illumination consists of two LED lamps. The frames were made of white acrylic plates, which do not affect the color valance of the illumination. b – d: The cleaning brush consists of three brushes and a servomotor, and is attached to the back of the knife holder by magnets (black arrows). e, f: Covers are used to stabilize the chamber temperature and avoid fogging. The cover has hollows for imaging the block-face, and inserting the illuminating device and the wire of the cleaning brush. The clear acryl plate allows checking of the condition of the block-face during imaging. Square pattern: 1 cm.

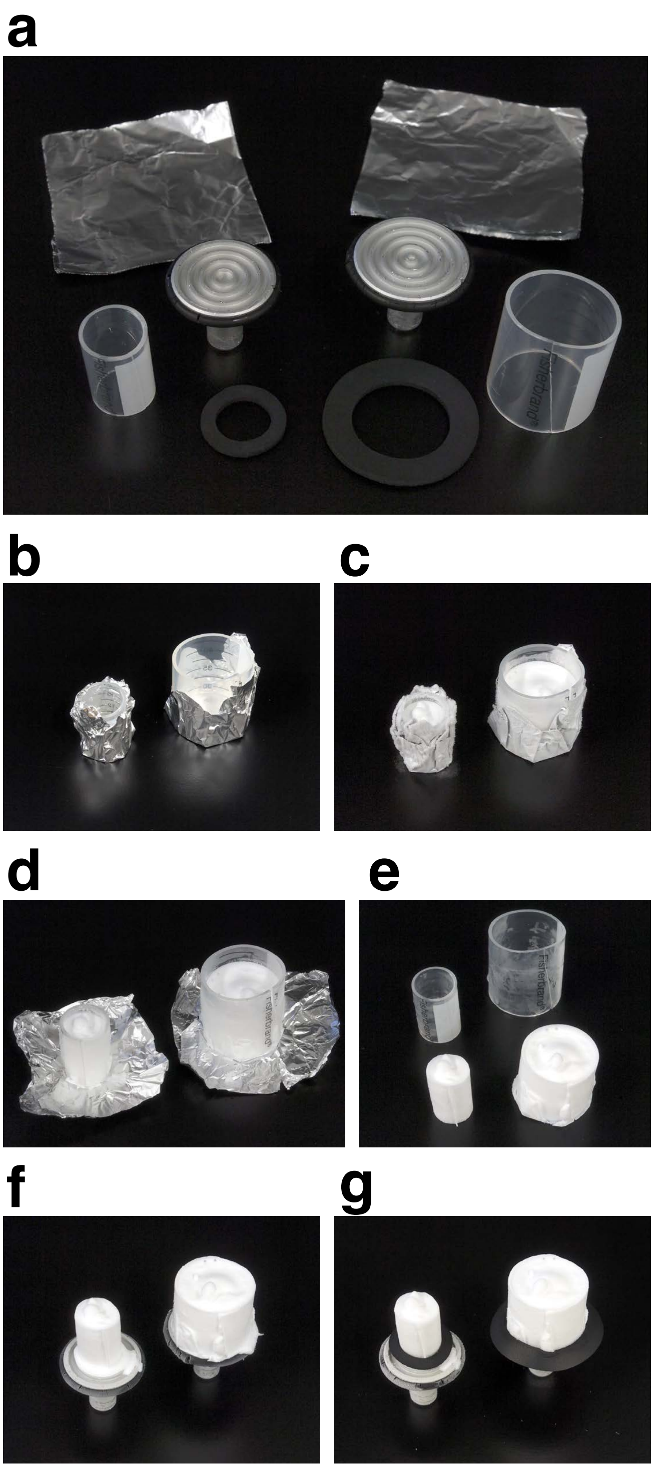

Supplementary Figure S5 | Procedure for making the frozen blocks with a right cylindrical shape and black background. a: Pieces of 15-ml or 50-ml plastic tube 25 mm in height, aluminum foil, black rubber rings, and specimen holders are used to make frozen blocks. The plastic tubes are cut open at one potion. b: The cups are made using plastic tubes and aluminum foil. c: The blocks are quickly frozen in liquid nitrogen. d, e: Aluminum foil and plastic tubes are removed. f: The frozen blocks are attached to the specimen holder. g: The black rubber rings are installed at the bottom of the block.

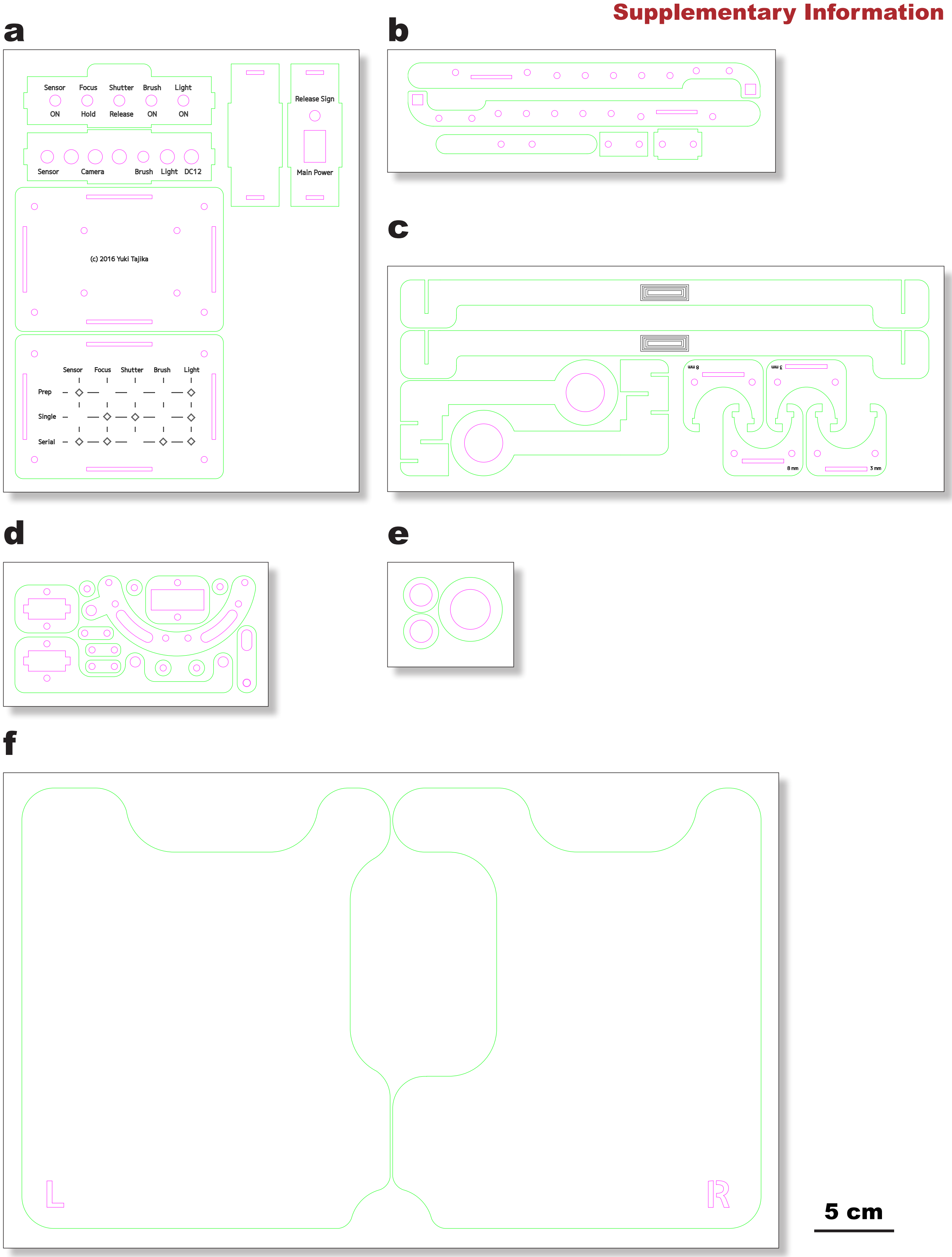

Supplementary Figure S6 | Drawings for self-made devices. The acrylic plates 2 mm thick were processed by a laser cutting machine to produce the enclosure of the controller (a), frames of the sensor (b), illumination (c), cleaning brush (d), and cover (e). A chloroprene sponge sheet 1 mm thick was processed to make the rubber rings (f). Lines indicate scratching pattern (black), first cutting lines (magenta), and second cutting lines (green).Drawings were saved as SVG files with a resolution of 90 dpi.

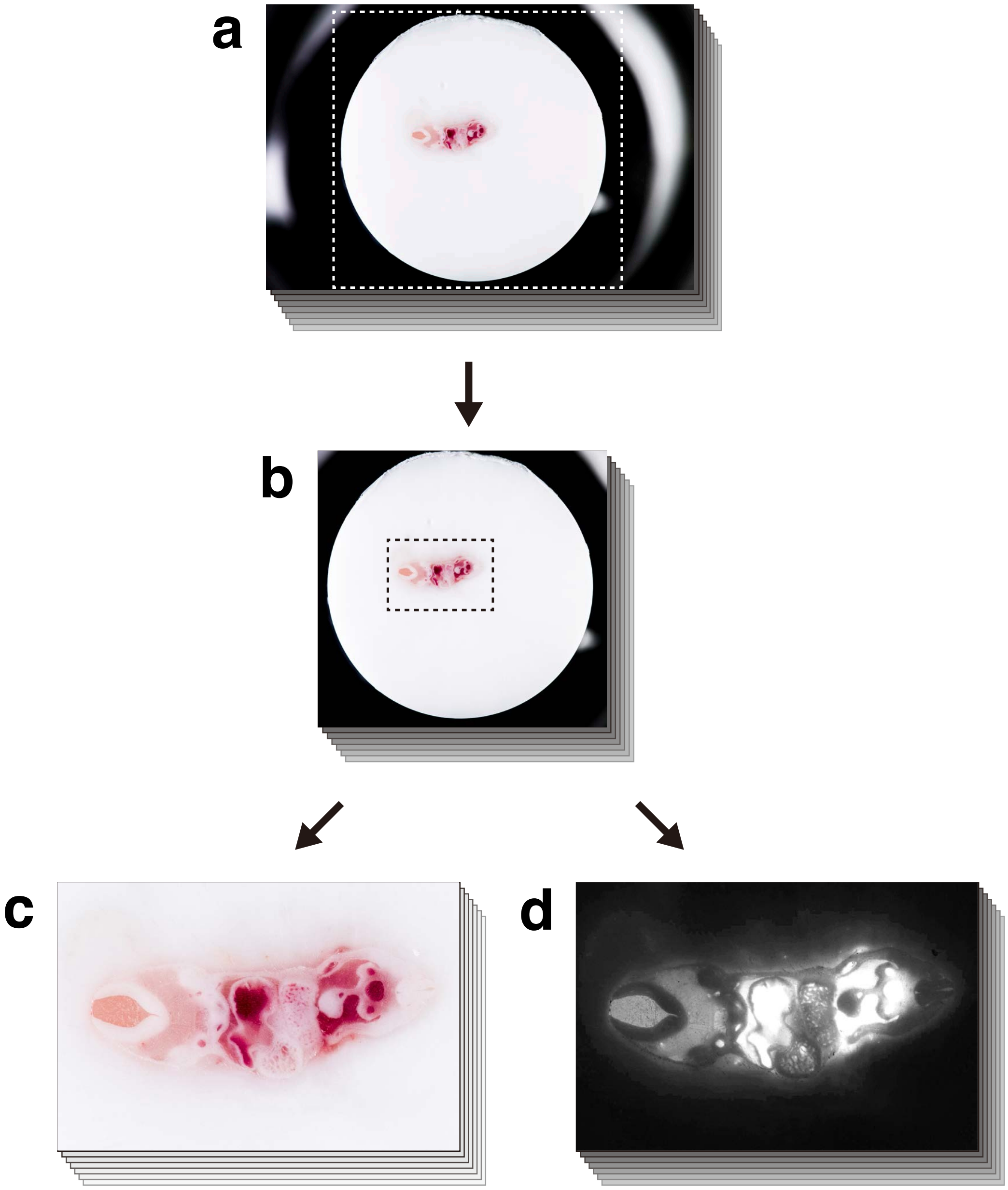

Supplementary Figure S7 | Procedure for image processing. a: Block-face images in RAW format are converted to JPEG images and cropped (boxed area). The outer circumference of the block is necessary for subsequent image registration. b: Images are registered, then cropped again (boxed area) to create a derivative image series. c, d: Color and grayscale image series are created. These series are converted to DICOM images and used for MPR and VR, respectively.

Supplementary Note | Codes for releasing the shutter (left) and regulating the cleaning brush (right)

```
//*************************************************************
// Designed for the sensor device, a part of the CoMBI system.
// ATtiny85 syncs cryostat handle and shutter release,
// using a photoreflector (Rohm RPR-220) and an optocoupler (Toshiba TLP785GB).
// Upload the code with the internal clock 1 MHz.
//*************************************************************

const int ledPin = 1;          //connect LED to 1
const int shutterPin = 0;      //connect an optocoupler to 0
const int sensorPin = A2;      //connect the sensor signal to A2
int sensorVal = 0;

void setup() {
    pinMode(ledPin, OUTPUT);
    pinMode(shutterPin, OUTPUT);
    digitalWrite(ledPin, HIGH);          // Twincle LED to sign turning on
    delay(200);
    digitalWrite(ledPin, LOW);
    delay(200);
    digitalWrite(ledPin, HIGH);
    delay(200);
    digitalWrite(ledPin, LOW);
}

void loop() {
  sensorVal = analogRead(sensorPin);
  delay(2);

  if (sensorVal > 250){               // If the sensor detect the light absorbing sheet
    digitalWrite(shutterPin, HIGH);      // Release shutter
    delay(10);
    digitalWrite(shutterPin, LOW);
    digitalWrite(ledPin, HIGH); // Indicate the sheet position and shutter release by LED
    delay(2000);
    digitalWrite(ledPin, LOW);
    }

    else{
      digitalWrite(ledPin, LOW);
      digitalWrite(shutterPin, LOW);
    }
}
```

```
//*************************************************************
// Designed for the cleaning device, a part of the CoMBI system.
// ATtiny85 controlls a Micro Servo SG92R.
//
// Download and Install "SoftwareServo.h" into the Arduino Software.
// Modify the SoftwareServo.h file to use <Arduino.h> instead of <WProgram.h>.
// Upload the code with the internal clock 1 MHz.
//
// (In case of Arduino/Genuino Uno, use "Servo.h".)
//*************************************************************

#include <SoftwareServo.h>

SoftwareServo myservo;  // create servo object

void setup()
{
  myservo.attach(0);  // attach the servo on pin 0 to the servo object
}

void loop()
{
  myservo.write(5);            // sets the servo position 5
  delay(300);                  // waits for the servo to get there
  SoftwareServo::refresh();    // recommended

  myservo.write(55);           // sets the servo position 55
  delay(300);
  SoftwareServo::refresh();

}
```
